# Neutrophil to high-density lipoprotein cholesterol ratio as the risk mark in patients with type 2 diabetes combined with acute coronary syndrome: a cross-sectional study

**DOI:** 10.1038/s41598-023-35050-6

**Published:** 2023-05-15

**Authors:** Hao Ren, Botao Zhu, Zhenyu Zhao, Yuan Li, Guiyuan Deng, Zewei Wang, Boyan Ma, Yuxin Feng, Zaiqiu Zhang, Xiaoxuan Zhao, Md Sayed Ali Sheikh, Ke Xia

**Affiliations:** 1 Department of Cardiology, Xiangya Hospital, Central South University, No. 87 Xiangya Road, Changsha, 410078 China; 2 XiangYa School of Medicine, Central South University, Changsha, China; 3 The Institute of Clinical Pharmacology, Xiangya Hospital, Central South University, No. 87 Xiangya Road, Changsha, 410078 China; 4 Department of Cardiology, Sun Yat-sen Memorial Hospital, Sun Yat-sen University, Guangzhou, China; 5 Internal Medicine Department, Cardiology, College of Medicine, Jouf University, 2014 Sakaka, Aljouf Saudi Arabia

**Keywords:** Acute coronary syndromes, Biomarkers

## Abstract

Chronic inflammation and dyslipidemia are important risk factors in developing atherosclerotic cardiovascular disease, such as coronary heart disease. Acute coronary syndrome (ACS) is one of the most dangerous syndromes in coronary heart disease. Type 2 diabetes mellitus (T2DM) is considered equal to coronary heart disease owing to the high cardiac risk induced by chronic inflammation and dyslipidemia. The neutrophil to high-density lipoprotein cholesterol ratio (NHR) is a novel and straightforward marker that reflects inflammation and lipid metabolic disorder. However, few studies have been on the role of NHR in assessing the risk of ACS in T2DM patients. Here we analyzed NHR level in ACS patients with T2DM, exploring its predictive and diagnostic values. 211 hospitalized ACS patients with T2DM were recruited as the case group, and 168 hospitalized T2DM patients as the control group (all patients collected from 6/2020 to 12/2021 in Xiangya Hospital). Biochemical test results and echocardiograms, as well as demographic information such as age, BMI, diabetes mellitus, smoking, drinking, and history of hypertension, were recorded. Frequencies, percentages, means, and standard deviations were used to describe the data. The shapiro–Wilk test was used to assess the normality of the data. Normally distributed data were compared using the independent sample T-test, and non-normally distributed data were compared using Mann–Whitney U test. Correlation analysis was performed using the Spearman rank correlation test, and receiver operating characteristic (ROC) curve analysis and multivariable logistic regression analysis were performed by SPSS version 24.0 (SPSS Inc) and GraphPad Prism 9.0 (GraphPad Software Inc). *p* < 0.05 was considered significant. In the study population, NHR was higher in patients with T2DM combined with ACS than in T2DM patients without ACS (*p* < 0.001). After adjusting for BMI, alcohol consumption, and history of hypertension, multifactorial logistic regression analysis revealed that NHR is a risk factor for T2DM patients combined with ACS (OR 1.221, *p* = 0.0126). Correlation analysis on all ACS patients with T2DM showed that NHR level was positively correlated with cTnI (r = 0.437, *p* < 0.001), CK (r = 0.258, *p* = 0.001), CK-Mb (r = 0.447, *p* < 0.001), LDH (r = 384, *p* < 0.001), Mb (r = 0.320, *p* < 0.001), LA (r = 0.168, *p* = 0.042) and LV levels (r = 0.283, *p* = 0.001). And meanwhile, NHR level was negatively correlated with EF (r = − 0.327, *p* < 0.001) and FS levels (r = − 0.347, *p* < 0.001). ROC curve analysis showed that NHR ≧ 4.32 had a sensitivity of 65.45% and a specificity of 66.19% for predicting ACS in T2DM patients [area under the curve (AUC) = 0.722, *p* < 0.001]. Furthermore, in all ACS patients with T2DM, the diagnostic power of NHR was stronger in ST-segment elevated ACS patients (STE-ACS) than that in non-ST-segment elevated ACS patients (NSTE-ACS) (*p* < 0.001). With its convenience and effective character, NHR could be a potential and new marker for predicting the presence, progression, and severity of ACS in T2DM population.

## Introduction

Type 2 diabetes mellitus (T2DM) is becoming a significant public health problem and a hot spot for clinical research with the rising incidence of it. The prevalence of diabetes in the Chinese adult population is about 11.6%, and the prevalence of prediabetes is around 50.1%^1^. As comorbidity of T2DM, cardiovascular disease (CVD) is also increasing nowadays^2,3^. A study has shown that CVD is the leading cause of death in patients with T2DM, accounting for approximately half of all deaths during the study period^4^. Acute coronary syndrome (ACS), one of the most severe types of coronary artery disease (CAD), is the most common CVD and is widely interrelated with T2DM^5^. ACS is triggered by the rupture of unstable atheromatous plaque in the coronary artery, resulting in thrombosis and coronary artery obstruction. Varying degrees of coronary stenosis bring varying degrees of myocardial ischemia^6^ and different symptoms. Non-ST-segment elevation ACS (NSTE-ACS) is characterized by the absence of significant ST-segment elevation on ECG. It is caused by partial or intermittent coronary arterial occlusion, which accounts for approximately 70% of ACS. ST-segment elevation ACS (STE-ACS) caused by complete coronary artery occlusion accounts for approximately 30% of ACS^7^. Commonly, many T2DM patients have ACS, which significantly increases the risk of major adverse cardiac events (MACE)^4,8^. Finding a relevant biomarker and identifying the ACS patients in T2DM might be of great benefit in preventing disease aggravation.

Studies have shown that inflammation and dyslipidemia play a key role in plaque formation and atherosclerosis progress, which leads to ACS^9,10^. Neutrophils, one of the markers that reflect inflammation, also participate in plaque instability and in the early formation of atherosclerosis^11,12^. In contrast, high-density lipoprotein cholesterol (HDL-C) is considered a protective factor against atherosclerosis due to its role in reversing cholesterol transport and its antioxidant ability^13^. Furthermore, HDL has been shown to regulate neutrophil activation and reduce neutrophil proliferation and migration^14^. HDL has been shown to regulate neutrophil activation and reduce neutrophil proliferation and migration^15^. The neutrophil to HDL-cholesterol ratio (NHR) is a composite marker reflecting inflammation and lipid metabolism. NHR is a strong predictor of cardiovascular disease in many studies^16–18^. Even though these two hematological parameters are inexpensive and readily available, the critical value of NHR in ACS patients has yet to be noticed or emphasized. This research aimed to assess the predictive and diagnostic value of NHR on the risk of developing ACS in T2DM patients.

## Materials and methods

### Study participants

**Figure 1:**
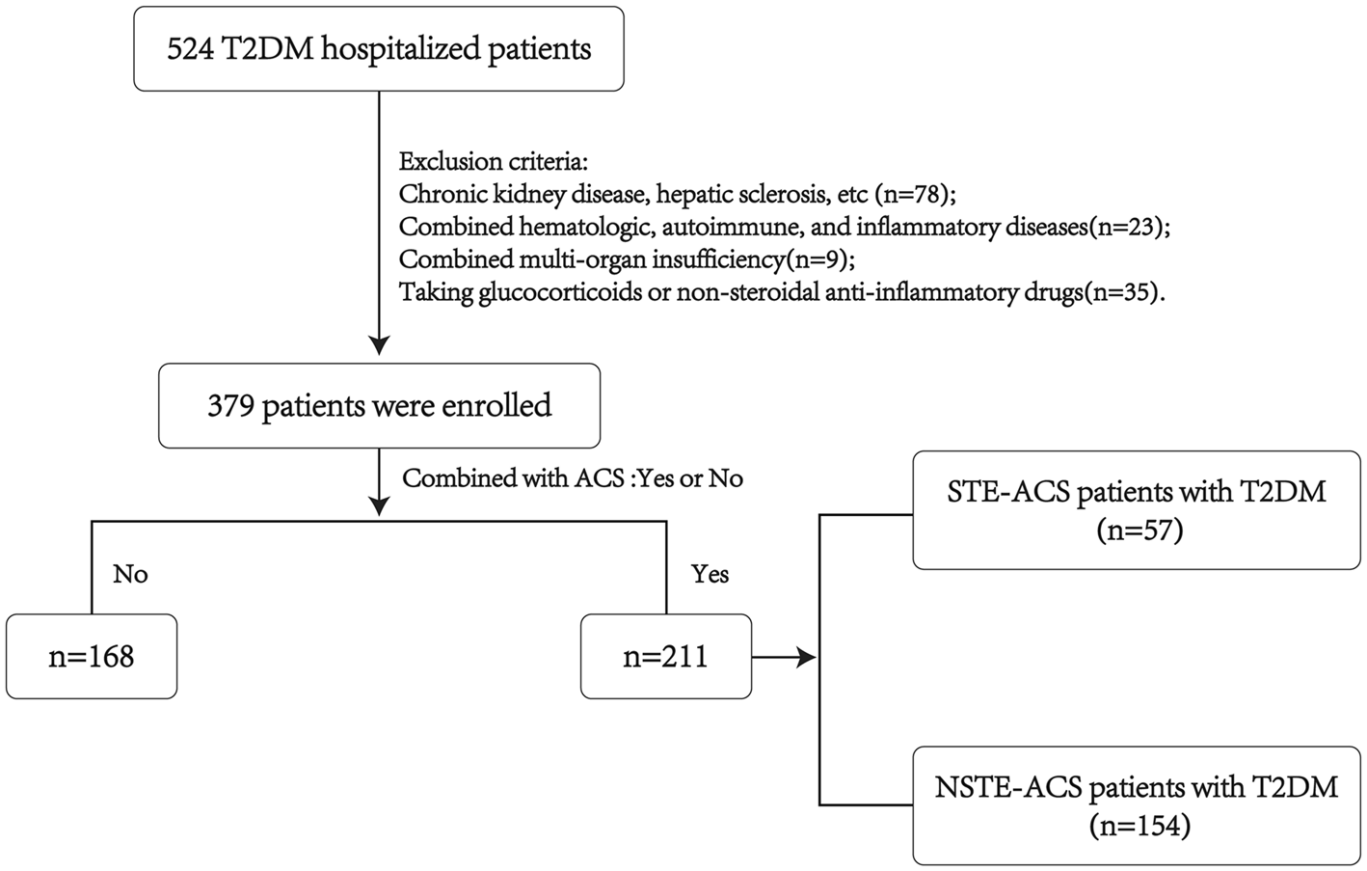
Flowchart of the enrolled patients. 524 adult T2DM patients were tested in this study, and at last, 379 enrolled patients were divided into two groups based on whether they combined with ACS. Demographic data and clinical and laboratory parameters were compared between these two groups.

Hospitalized patients diagnosed with T2DM at Xiangya Hospital of Central South University from June 2020 to December 2021 were enrolled. T2DM patients combined with ACS, including STE-ACS and NSTE-ACS study, were treated as the study population, and T2DM patients without ACS were treated as a control population. This cross-sectional study was performed per the ethical guidelines of the Declaration of Helsinki and was approved by the Medical Ethics Committee of Xiangya Hospital of Central South University. Written informed consent was obtained from all subjects before enrollment. ACS was defined as (1) clinical manifestations, (2) elevated cTnI, (3) elevated cardiac enzymes^19^, and (4) electrocardiographic changes. Exclusion criteria included: chronic kidney disease with serum creatinine > 2.5 mg/dL, hepatic sclerosis, congestive heart failure, chronic lung disease, symptomatic peripheral vascular disease, tumors, and chronic infections; those with combined hematologic, autoimmune, and inflammatory diseases; those with combined multi-organ insufficiency; and those taking glucocorticoids or non-steroidal anti-inflammatory drugs (Fig. 1).

### Clinical and biochemical parameters

Information such as age, sex, body mass index (BMI), smoking history, drinking history, history of hypertension, and parameters such as RBC, Hb, PLT, Glu, TG, TC, HDL-C, LDL-C, UA, Urea, Cre, CK, CK Mb, LDH, Mb, and NHR were all collected. The levels of hemoglobin, red blood cells, and platelets were measured based on the Kurt principle by the German BeckmanDxH800 blood analyzer. Glu, TG, TC, HDL-C, LDL-C, UA, Urea, Cre, CK, CK Mb, LDH, and Mb were measured in the spectrophotometric determination method by Germany BeckmanAU680 biochemical analyzer. The dimensions of the Left Ventricle (LV) and Left Atrial (LA) on the end-diastolic phase, the ejection fraction (EF), and the fractional shortening (FS) were performed by echocardiogram.

### Statistical analysis

SPSS 24.0 statistical software and GraphPad Prism 9.0 were used to analyze the data. Data are expressed as the mean ± standard deviation (SD) for continuous variables and as percentages for discreet variables. The normality of the data was assessed using the Shapiro–Wilk test. Comparison of normally distributed continuous variables between groups was performed by independent sample T-test. Data that did not have a normal distribution were expressed as medians (interquartile range). Mann–Whitney U test was used for comparison of variables that did not have a normal distribution. The data for categorical variables were analyzed by the χ^2^ test. Correlation between variables was determined by Spearman’s correlation test. Different logistic regression models were implemented to interrogate the association of ACS in patients with T2DM. In model 1, no covariates were adjusted; in model 2, BMI, smoking and history of hypertension were adjusted. The model was assessed using the Hosmer–Lemeshow test. And ROC curve analysis was used to evaluate the diagnostic value and to define the diagnostic cut-off value of NHR concentrations in the T2DM patients combined with ACS. A two-sided *p* value < 0.05 was considered to indicate statistical significance.

### Ethics approval and consent to participate

The study protocol was approved by the Medical Ethics Committee of Xiangya Hospital of Central South University and all methods were performed in accordance with the relevant guidelines and regulations.

## Results

### Baseline clinical characteristics and biochemical measurements

A total of 379 study subjects were included, including 211 cases in the T2DM combined with the ACS group and 168 cases in the T2DM group. Demographic, clinical, and biochemical data for the cases (T2DM with ACS) and controls (T2DM) were shown in Table 1. In comparing the general data of the 2 groups, the differences in age and history of hypertension were statistically significant (*p* < 0.05). Cases had statistically significantly higher NHR, CK, CK-Mb, LDH, Urea, and Cre levels than controls. Cases had statistically significantly lower serum LDL-C and TC levels than controls. The differences in sex, BMI, smoking history, drinking history, RBC, Hb, PLT, Glu, TG, HDL-C, and UA were not statistically significant (*p* > 0.05, Table 1).

**Table 1:** Clinical and biochemical properties of the study population.

Parameters	T2DM without ACS, controls (n = 168)	T2DM with ACS, cases (n = 211)	*p*-value
Male/female, N	103/65	143/68	0.190
Age/(years)	55 (48–63)	63 (54–70)	< 0.001
BMI/(kg m^-2^)	24.61 ± 2.5	24.01 ± 3.22	0.167
Smoking, N (%)	38 (22.62%)	63 (29.86%)	0.120
Drinking, N (%)	30 (17.86%)	36 (17.06%)	0.839
History of hypertension, N (%)	77 (45.83%)	142 (67.30%)	< 0.001
RBC/(× 10^12^ L^−1^)	4.36 (3.99–4.81)	4.26 (3.82–4.72)	0.115
Hb/(g L^−1^)	133.00 (119.00–144.25)	132.00 (117.00–144.00)	0.441
PLT/ (× 10^9^ L^−1^)	211.53 ± 66.99	211.12 ± 74.35	0.955
Glu/(mmol L^−1^)	6.72 (5.52–8.38)	7.17 (5.86–9.08)	0.182
TG/(mmol L^−1^)	1.831.16–2.80)	1.72(1.17–2.16)	0.122
TC/(mmol L^−1^)	4.814.02–5.50)	4.06(3.39–5.09)	< 0.001
HDL-C/(mmol L^−1^)	0.97 (0.85–1.16)	0.97(0.81–1.09)	0.212
LDL-C/(mmol L^−1^)	3.13 ± 0.87	2.72 ± 0.84	< 0.001
UA/(mmol L^−1^)	338.30 (275.70–392.70)	356.10 (287.20–429.93)	0.068
Urea/(mmol L^−1^)	5.61 (4.49–7.48)	6.20 (4.75–8.69)	0.027
Cre/(mmol L^−1^)	79.00 (65.00–94.00)	86.10 (73.90–110.23)	< 0.001
CK/(U L^−1^)	80.20 (60.05–120.58)	99.60 (60.30–217.20)	0.004
CK-Mb/(U L^−1^)	12.00 (10.00–14.35)	14.80 (11.50–23.20)	< 0.001
LDH/(U L^−1^)	177.50 (153.55–208.00)	219.00 (173.90–297.00)	< 0.001
Mb/(U L^−1^)	33.25 (21.43–50.35)	36.00 (22.90–77.40)	0.042
NHR	3.62 (2.69–5.13)	5.00 (3.65–6.88)	< 0.001

*BMI* body mass index, *RBC* red blood cell, *PLT* platelet, *Hb* hemoglobin, *Glu* blood glucose, *TG* triglyceride, *TC* total cholesterol, *HDL-C* high-density lipoprotein cholesterol, *LDL-C* low-density lipoprotein cholesterol, *UA* uric acid, Cre creatinine, *CK* creatine kinase, *CK-MB* creatine kinase-MB, *LDH* lactate dehydrogenase.

### Multi‑logistic regression analysis to determine the relationship between the NHR and the presence or absence of ACS in T2DM patients

**Figure 2:**
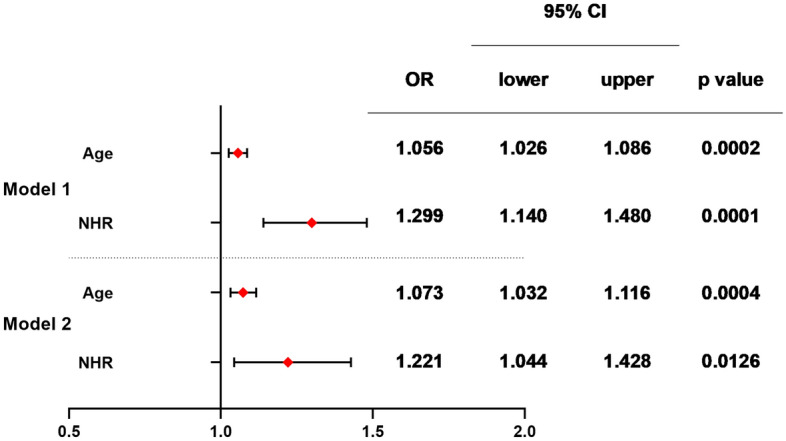
Multivariable Logistic regression analysis to identify variables that were independently correlated with the presence or absence of ACS in T2MD patients. *OR* odds ratio, *CI* confidence interval. Model 1: Unadjusted. Model 2: Adjusted for BMI, drinking and history of hypertension. n = 379.

In this cross-sectional study, the variables we included in the multivariable logistic regression analysis, including NHR, age, TC, LDL-C, UA, Urea, CK, CK-Mb, and LDH, which were statistically significant (*p* < 0.05) in the univariate logistic regression analysis. In the unadjusted model, higher NHR and age were independent risk factors for developing ACS in patients with T2DM (Model 1, Hosmer–Lemeshow test *p* = 0.519). Further, multivariable logistic regression analysis conducted in the 379 patients after adjustments for BMI, drinking, and history of hypertension suggested that higher NHR and age were independent risk factors of ACS in T2DM patients (Model 2, Hosmer–Lemeshow test *p* = 0.793) (Fig. 2).

### Correlation between NHR level and clinical parameters in T2DM patients with ACS

**Figure 3:**
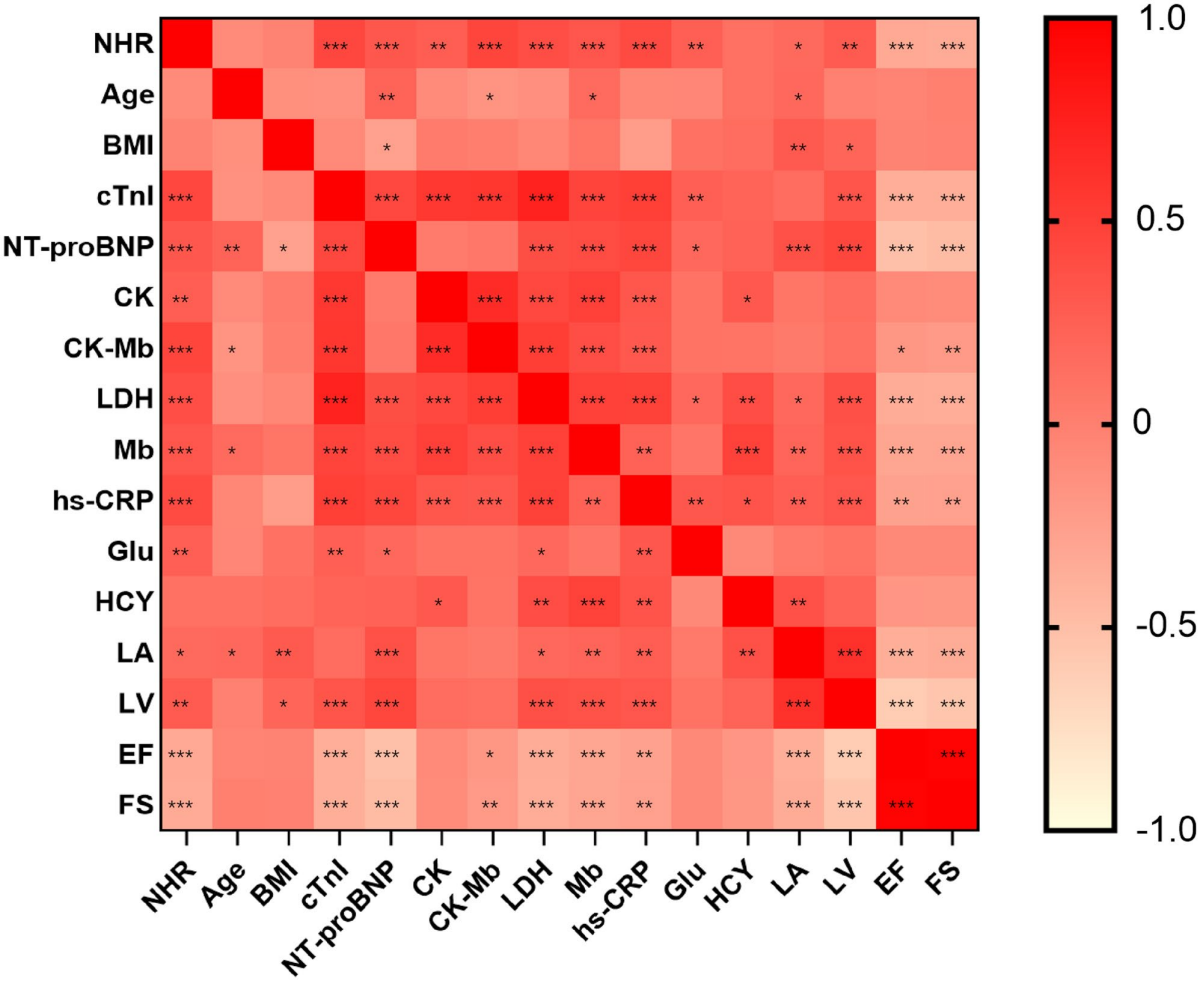
Heat map of correlation of variables in T2DM patients with ACS.

Correlation analyses undertaken on all T2DM patients with ACS showed that NHR level was positively correlated with cTnI (r = 0.437, *p* < 0.001), CK (r = 0.258, *p* < 0.001), CK-Mb (r = 0.447, *p* < 0.001), LDH (r = 384, *p* < 0.001), Mb (r = 0.320, *p* < 0.001), LA (r = 0.168, *p* = 0.042) and LV levels (r = 0.283, *p* = 0.001) and negatively correlated with EF (r = − 0.327, *p* < 0.001) and FS levels (r = − 0.347, *p* < 0.001, Table 2, Fig. 3(*p < 0.05, **p < 0.01 and ***p < 0.001)).

**Table 2:** Correlation between NHR and other variables in T2DM patients with ACS.

Parameters	NHR	
	r value	*p*-value
Age	− 0.096	0.222
BMI	− 0.035	0.745
cTnI	0.437	< 0.001
NT-proBNP	0.305	< 0.001
CK	0.258	0.001
CK-Mb	0.447	< 0.001
LDH	0.384	< 0.001
Mb	0.320	< 0.001
hs-CRP	0.410	< 0.001
Glu	0.250	0.002
HCY	0.119	0.354
LA	0.168	0.042
LV	0.283	0.001
EF	− 0.327	< 0.001
FS	− 0.347	< 0.001

*cTnI* cardiac troponin I, *NT-proBNP* N-terminal pro-B-type natriuretic peptide, *hs-CRP* hypersensitive C-reactive protein, *HCY* homocysteine, *LA* left atrium, *LV* left ventricle, *EF* ejection fractions, *FS* fractional shortening.

### NHR levels in groups

**Figure 4:**
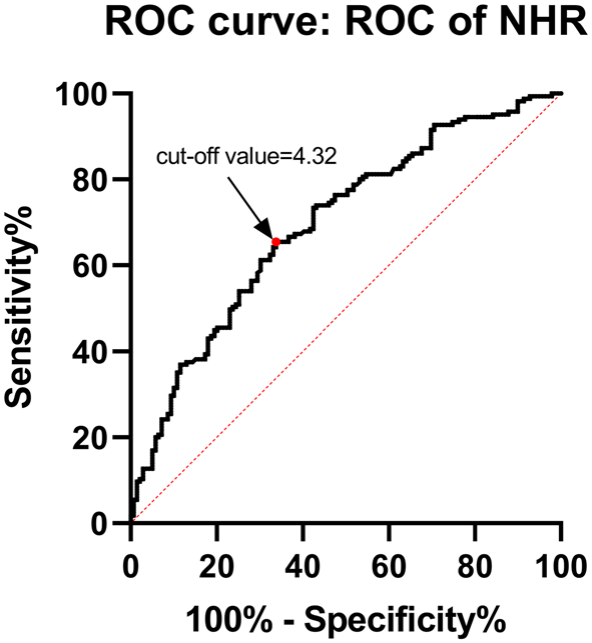
ROC curve for NHR discriminatory abilities towards ACS in patients with T2DM.

NHR level was statistically significantly higher in study cases as compared to controls [5.00 (3.65–6.88) vs 3.62 (2.69–5.13), *p* < 0.001]. Utilizing the ROC curve for the value of NHR and risk of ACS in patients with T2DM, the greatest increase in the ACS risk was seen at NHR levels of more than 4.32 [Fig. 4, AUC = 0.696, sensitivity of 65.45% (95% CI 57.61% to 72.57%) and specificity of 66.19% (95% CI 57.61% to 73.85%), Youden’s index = 0.316, *p* < 0.001].

The study included 211 T2DM patients combined with ACS, including 57 patients with STE-ACS. Of those 57 patients, 52 patients (91.23%) were identified by the above cutoff value (cutoff value = 4.32). In addition, there were 154 patients with NSTE-ACS and 100 NSTE-ACS patients (64.94%) were identified by it, which suggested that the NHR level has a significant difference between the above two sub groups in ACS patients combined with T2DM (Table 3, *p* < 0.001).

**Table 3:** Identification rate of different ACS types in T2DM patients by cut-off values.

ACS types	Total cases	Identified cases	Rate (%)	*p* value
NSTE-ACS	154	100	64.94	< 0.001
STE-ACS	57	52	91.23	

## Discussion

In this study, we demonstrated that NHR is an independent risk factor for the development of ACS in the T2DM population. Our result showed that NHR had a significant positive correlation with some biochemical markers of myocardial ischemia, necrosis, and remodeling in T2DM patients combined with ACS. It has strongly suggested the potential importance of NHR in the progression of ACS in T2DM patients. Furthermore, our study showed that NHR≧4.32 has good discriminatory power in diagnosing ACS patients in the T2DM population, although the accuracy may need to be demonstrated by more patients and data. NHR may help predict ACS patients in T2DM patients.

With further analysis, we found the correlation of NHR with biochemical marks of myocardial ischemia, necrosis, and remodeling (cardiac ultrasound indices) in T2DM patients combined with ACS. The results revealed that NHR levels were significantly and positively correlated with cTnI, CK, CK-Mb, LDH, and MB, which are indicators of myocardial ischemia and injury. In addition, NHR levels also showed a significant positive correlation with left atria (LA) and left ventricle (LV) levels, suggesting a relationship between NHR and cardiac remodeling aspects. NHR levels were significantly and negatively correlated with EF and FS, indicators of cardiac function. It has been shown that neutrophils are a critical contributor to LV infarct wall thinning due to cardiac remodeling and that higher neutrophils correspond to lower EF and FS^20^. The correlation of these markers may explain their diagnostic value in T2DM patients combined with ACS.

To evaluate the diagnostic and predictive value of NHR level for ACS in T2DM patients, we performed ROC analysis to determine the cut-off value, sensitivity, specificity, AUC, and Youden’s index of NHR for diagnosing acute coronary syndrome in T2DM patients. Further, we examined the discriminatory ability of the cut-off value of NHR in T2DM patients combined with STE-ACS and NSTE-ACS. Our results showed that NHR had higher discriminatory accuracy and diagnostic value in T2DM patients combined with STE-ACS, which is of higher severity than NSTE-ACS. This suggests that NHR may correlate with the severity of ACS in T2DM patients. Traditionally, the diagnosis of ACS has been made by clinical symptoms, ECG changes, cardiac biomarkers, and, in some cases, cardiac imaging. However, the search for new biomarkers is increasing, which will help to improve the diagnosis and prognosis of patients with ACS. Our study suggests that NHR levels could be potentially used as a new complementary marker, with the traditional cardiac markers together, for diagnosing ACS patients in the T2DM population.

Recently, an increasing number of studies have used composite predictors of hematological parameters as novel potential risk markers. The ratios of different parameters can provide more comprehensive information than traditional single parameters and have considerable diagnostic and predictive power^16–18,21–23^. For example, neutrophil to HDL-C ratio (NHR)^16–18,23^, platelet to lymphocyte ratio (PLR)^24^, monocyte to HDL-C ratio (MHR)^21,22^, lymphocyte to HDL-C ratio (LHR)^25^, platelet to HDL-C ratio (PHR)^26^, and triglyceride to HDL-C ratio (THR)^27^. These markers from routine blood tests have a good future due to their affordability and accessibility.

Many previous studies have found that NHR has a better application in the above-mentioned indices due to its unique advantages^16–18,23^. Firstly, T2DM and ACS involve complex pathological processes of inflammatory response and abnormal lipid metabolism^9,10,28^. NHR can not only present both inflammatory state and lipid metabolism, but also indicate the interaction between neutrophils and HDL-C. Advanced atherosclerotic plaques have a high number of neutrophils and their counts are positively correlated with the histopathological features of rupture-prone atherosclerotic lesions^29^. It has also been demonstrated that low HDL-C is an important factor in accelerating atherosclerosis in diabetic patients^30^. Neutrophils and HDL-C play a mutually regulating role in ACS. Activated neutrophils can also mediate HDL oxidation and impair cholesterol efflux by possessing enzymes that produce oxidants^31^. In contrast, HDL-C can inhibit neutrophil activation, adhesion, proliferation and migration^15^. Secondly, neutrophils make up the major part of leukocytes and therefore they can better reflect cardiovascular risk than monocytes and lymphocytes^11,12,32,33^. On the other hand, neutrophils are considered to be the primary players in the acute inflammatory response and play an important role in the subsequent activation of monocytes and lymphocytes^34–36^. These key roles of neutrophils allow NHR to provide better diagnostic and predictive value for cardiovascular disease.

However, our study still has some limitations that should be noted. First, the NHR was measured only once at baseline, which may not reflect the time-dependent association of dynamic changes in NHR with clinical performance. Therefore, our study can only demonstrate that NHR is helpful in the diagnosis and prediction of ACS in patients with T2DM, and more data and more diverse studies are needed to support our view. Second, although the study process was adjusted for covariates as much as possible, we cannot exclude possible residual confounding effects of unmeasured or unincluded variables, such as the different disease duration and different treatments for each T2DM patient. Third, variables such as lifestyle factors and medical history were self-reported, which could lead to recall bias. Finally, this was a cross-sectional study, so a causal relationship between NHR and the development of ACS in T2DM patients could not be demonstrated. Further prospective studies are needed to analyze whether lowering NHR reduces the occurrence and progression of ACS.

## Conclusion

In conclusion, this study suggests that elevated NHR has great potential to be a convenient and effective measure to predict the presence and progression of ACS in T2DM patients. Although more investigations, especially longitudinal ones, are still needed to further validate this.

## Data Availability

The datasets used and/or analysed during the current study available from the corresponding author on reasonable request.
